# Influence of Various Excitation Parameters on Polymer Flow Properties in Twin-Screw Extruders Simulated with Smoothed Particle Hydrodynamics

**DOI:** 10.3390/polym18030360

**Published:** 2026-01-29

**Authors:** Tianlei Liu, Hesheng Liu, Tianwen Dong, Jiamei Lai, Wei Yu, Zhong Yu, Huiwen Yu

**Affiliations:** 1 Jiangxi Key Laboratory of High-Performance Precision Molding, Nanchang University, Nanchang 330031, China; liusya0618@163.com (T.L.); laijm@163.com (J.L.); 352400240017@email.ncu.edu.cn (W.Y.); 2 School of Mechatronics and Vehicle Engineering, East China Jiao Tong University, Nanchang 330013, China; 3 Jiangxi Province Key Laboratory of Applied Optical Technology, Shangrao Normal University, Shangrao 334001, China; 306150@sru.edu.cn; 4 School of Mechanical and Automation Engineering, Wuyi University, Jiangmen 529020, China; ddlianyi@163.com

**Keywords:** vibration-assisted forming, particle distribution, crossover degree, SPH

## Abstract

Vibration-assisted technology has been employed to satisfy various requirements for different polymeric products due to its excellent performance, but because of the large inertia of the vibration excitation system, these attempts are strictly limited to several fixed vibration amplitudes and frequencies in small extruders or injectors. The purpose of this study is to carry out a numerical investigation via smoothed particle hydrodynamics (SPH) and to perform a comparative analysis of physical parameters among different cases from various perspectives on the fluid channel in twin-screw extruders (TSEs). The results demonstrate that certain combinations of larger vibration amplitudes and frequencies can significantly enhance the velocity, pressure, and particle distribution characteristics within the flow channel. However, no monotonic (i.e., strictly increasing or decreasing) trends are observed with respect to either amplitude or frequency alone. These findings are in excellent agreement with previously reported experimental studies and confirm that the meshless smoothed particle hydrodynamics (SPH) method is a robust and effective computational tool for investigating how various vibrational parameters influence flow behavior in twin-screw extruders (TSEs). Moreover, the results underscore that optimal amplitude and frequency selections must be tailored to the specific rheological and thermal properties of the polymer being processed. This work establishes a solid theoretical and numerical foundation for integrating superimposed vibration-assisted technology into the design optimization of TSE systems.

## 1. Introduction

The performance requirements of polymeric products are getting higher and higher with the development of science and technology, and conventional single-screw extruders or injectors cannot meet the application needs due to poor mixing capacity [1,2]. In order to solve this problem, many researchers have improved screw structures according to the characteristics of different materials and developed a lot of new single screws, such as barrier screws [3], pin screws [4], and wave screws [5]. These new screws greatly improve the dispersive mixing capacity of the polymer processing device and can meet many application requirements [6,7,8,9].

However, because new screws are mostly developed for specific materials, this brings many problems: narrow application, high manufacturing cost, leakage, cleaning difficulties, and so on [1]. In order to address these problems, some researchers have continued to improve structural designs and have developed twin screw [10], tri-screw [11,12] and other multi-screw [13] devices that have two or more screws placed side by side in the barrel. These screws can squeeze and drag the polymer material several times when the polymer material passes through the engagement zones between screws, and this new feature can enhance the mixing and homogenization effect. At the same time, the screws are able to remain nested with each other during the rotation process, and this is conducive to cleaning the material in each screw’s grooves so as to achieve a self-cleaning effect [1,14,15]. Furthermore, the mixing capacity and processing application range of multi-screw devices can be significantly improved by setting different combinations of kneading blocks on the screws [16].

Besides improving mechanical structure, some researchers have introduced a vibration force field into polymer processing devices and have developed various vibration-assisted technologies, such as ultrasonic plasticization, oscillation pressure injection molding and vibration-assisted injection molding [17,18,19,20,21]. Some researchers have introduced vibrations to the die part at the head of extruders and to the injection molding process and have made many remarkable achievements. Under vibration-assisted processing conditions, the mechanical properties of the extrudate can be substantially improved by increases of 150% in elongation at fracture and 70% in traction within a given range of the amplitude–frequency of oscillations [22]. Testing results on material extrusion parts [23] show that applied vibration can reduce a part’s defects of porosity, inclusion and separation between layers and thus improve bonding strength. In producing carbon-fiber-reinforced polymer composites [24], ultrasonic vibration can lead to a substantial increase of up to 16% in fiber volume fraction, 20% in flexural strength and 90% in fracture energy, and the vibration can also lead to a decrease in apparent viscosity in the extrusion melt [25]. Experiments on vibration-assisted injection molding show that the tensile strength of a polymer sample can be increased from 43.9 MPa to 53.2 MPa [26], the filling time can be shortened from 2.15 to 1.95 s [20], and the cycle time can be reduced by 40% [21]. Then, researchers have changed the excitation position and introduced superimposed vibrations to the plasticization process of molten polymers. Results [27] show that head pressure can be reduced by 20–30%, and the yield per unit time can be increased by 1.4–2.0 times.

The above vibration applied to the local part of the extrusion equipment is beneficial for improving the performance of the product to some extent. However, the key factor affecting the performance of the product is the quality of plasticization, so the methods of loading the vibration on local areas of the whole extruder limit the effect of vibration technology. Therefore, researchers have begun to develop vibration equipment that can be applied to the entire process. One device of this type was first put forward and designed by Qu [28], namely, the electromagnetic dynamic plasticizing extruder. Compared with traditional single-screw extruders, the application of this technology has achieved a lot of remarkable results, such as a reduction in volume and weight by more than 45%, manufacturing costs by more than 45%, energy consumption by more than 35%, and noise down to less than 75 dB [29,30,31,32]. Furthermore, this vibration technology can also be applied to a tri-screw extruder, and experiments [1] show that the tensile strength and elongation at break are increased by 65.34% and 82.92%, respectively, when the vibration frequency is set to 10 Hz, and the amplitude is set to 1.0 mm.

The electromagnetic dynamic extruder, however, requires two sets of vibrating equipment to produce a vibration field in the axial and circumferential directions, which makes this method suitable only for small polymer processing devices due to the significant inertia of the whole plasticizing system. In order to solve this limitation of vibration technology, Liu [33] introduced differential drive technology into the screw drive components of polymer forming devices. This drive technology can keep the screw running in a sinusoidal way and drive the material in the screw groove to pulsate sinusoidally. This method can be realized only by transforming the drive component and has nothing to do with the inertia of the entire plasticizing system, so it can be applied to all sizes of polymer processing equipment. Numerical studies with this method on single-screw mixing components [34] and twin-screw mixing components [35] show that pulsation excitation can form a much stronger pressure field, which is quite conducive to the flow of particles, and can also produce a changing velocity field, which is beneficial for enhancing and weakening the flow field of polymers in turn, thus allowing for a dynamic state under pulsation excitation with a much better mixing performance relative to the steady state without a vibration pulsating field.

The research from the literature [35], however, has only been conducted with one group of specific vibration amplitudes and frequencies, so the induced conclusions have certain limitations, and it is difficult to determine whether that group of amplitudes and frequencies is suitable for actual manufacturing. In this paper, we expand our research on co-rotating twin-screw components with many more groups of amplitudes and frequencies. The open-source software DualSPHysics V5.0.1 is used as a platform for the smoothed particle hydrodynamics (SPH) method. Based on numerical simulation, the influence of different speed pulsation parameters on the physical field of the polymer flow in the twin-screw channel is studied in detail, aiming to provide more effective reference for the design and manufacture of an actual pulsating twin-screw extruder. The primary content of this paper can be outlined as follows: in Section 2, the models and methods used in this study are introduced in detail, including the geometric model, the governing equations, and the parameter setting; then, in Section 3, the results and discussion are presented contrastively with views of the whole channel, the clips, and the tracking particles with expanding cases; and finally, the results are obtained in Section 3, and the limitations and future prospects of the current research work are concluded in Section 4.

## 2. Materials and Methods

### 2.1. Governing Equations and SPH Algorithm

Mass and momentum conservation equations written in Lagrangian form are given by [36,37]

(1) $$\frac{d\rho }{dt}=-\rho \nabla \cdot v$$

(2) $$\frac{dv}{dt}=-\frac{1}{\rho }\nabla p+\frac{1}{\rho }\nabla \cdot \tau +F$$

where *ρ*, ***v***, *p* and ***F*** are the density, velocity vector, pressure and a body force per unit mass, respectively. ***τ*** is the shear tensor, and

(3) $$\tau =2\mu \varepsilon -\frac{2}{3}\mu (\nabla \cdot v)I$$

(4) $$\varepsilon =\frac{1}{2}[\nabla v+{\left(\nabla v\right)}^{T}]$$

where *μ* is the dynamic viscosity, ***ε*** is the strain rate tensor, and ***I*** is the unit vector.

The above two equations are built on a continuum, and some treatment is necessary to apply them in the SPH method. The main concept is that all objects in SPH can be represented by a finite number of particles carrying individual masses and occupying separate spaces [37]. Therefore, all functions describing various field parameters can be achieved by particle approximation, and a function, together with its spatial derivative, can be written as

(5) $$f(x_{i})=\sum_{j=1}^{N}\frac{m_{j}}{\rho_{j}}f(x_{j})\cdot W_{ij}$$

(6) $$\nabla \cdot f(x_{i})=-\sum_{j=1}^{N}\frac{m_{j}}{\rho_{j}}f(x_{j})\cdot \nabla W_{ij}$$

where

(7) $$W_{ij}=W(x_{i}-x_{j})$$

(8) $$\nabla_{i}W_{ij}=\frac{x_{i}-x_{j}}{r_{i}-r_{j}}\frac{\partial W_{ij}}{\partial r_{ij}}$$

**Figure 1:**
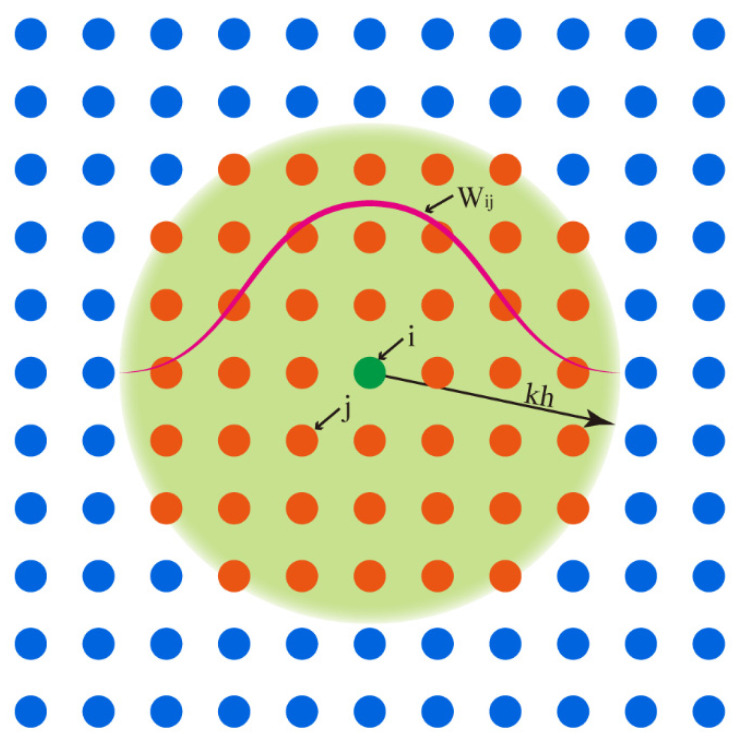
The support domain of the smoothing function and the problem domain.

Here, as shown in Figure 1, the problem domain is composed of particles (marked in blue) and the support domain (the circle marked in green and with a radius of *kh*; h is the smoothing length, and *k* is a constant related to the smoothing function) of one particle, *i* (marked in green), is also defined by particles (marked in orange), which are selected from particles composing the problem domain according to certain rules. *N* is the number of particles located in the support domain of particle *i*, $x_{i}$ and $x_{j}$ are the position of particles *i* and *j*, vectors $r_{i}$ and $r_{j}$ are displacement of particles *i* and *j* relative to the coordinate origin, $m_{j}$ and $\rho_{j}$ are the mass and density of particle *j*, and $W_{ij}$ is the smoothing function of particle *i* evaluated at particle *j*.

In this study, because the simulated object is a 3D model, the smoothing function in Equation (7) is selected in the Wendland form with the compact support of 1.2 *h*:

(9) $$W(R,h)=\alpha_{d}\times \left\{\begin{array}{ll} {\left(1-\frac{R}{2}\right)}^{4}\times (2R+1), & 0\leq R<1;\\ 0 & R\geq 2, \end{array}\right.$$

where $R=\left|r_{i}-r_{j}\right|/h$, and the normalization constant, $\alpha_{d}$, is equal to 21$/(16\pi h^{3})$.

The fluid simulated in this research is assumed to be of weak compressibility, so the weakly compressible SPH (WCSPH) and Delta-SPH algorithms are integrated to introduce a diffusive term to reduce the density fluctuations. Based on the above discussion, the discretized form of the mass and momentum conservation equations, Equations (1) and (2), can be transformed as [38,39,40]

(10) $$\frac{d\rho_{i}}{dt}=\sum_{j=1}^{N}m_{j}\cdot v_{ij}\cdot \nabla_{i}W_{ij}+2\xi hc\sum_{j=1}^{N}\left(\rho_{i}-\rho_{j}\right)\frac{r_{ij}\cdot \nabla_{i}W_{ij}}{{r_{ij}}^{2}+\eta h^{2}}\frac{m_{j}}{\rho_{j}}$$

(11) $$\frac{dv_{i}}{dt}=-\sum_{j=1}^{N}m_{j}\left(\frac{p_{i}}{\rho_{i}^{2}}+\frac{p_{j}}{\rho_{j}^{2}}\right)\nabla_{i}W_{ij}+\sum_{j=1}^{N}\frac{m_{j}(\mu_{i}+\mu_{j})r_{ij}\cdot \nabla_{i}W_{ij}}{\rho_{i}\rho_{j}({\left|r_{ij}\right|}^{2}+\eta h^{2})}v_{ij}+F$$

where the Delta-SPH diffusive coefficient *ξ* = 0.1 is used, *c* is the sound speed, $\rho_{i}$ is the density of particle *i*, $v_{i}$ is the velocity of particle *i*, and $\mu_{i}$ and $\mu_{i}$ are the dynamic viscosities of particle *i* and particle *j*, respectively. In order to ensure that the denominator is not zero when |*r_ij_*|^2^ equals zero during the numerical calculation, an infinitesimally small value is added to the denominator. This value is usually chosen to be related to the smooth length, *h*; here, a value of *ηh*^2^ is selected, and *η* is generally set to 0.01. $p_{i}$ and $p_{j}$ are the pressures for particle *i* and *j*, which can be calculated directly from the density based on an equation of state [41]:

(12) $$p=B\left[{\left(\frac{\rho }{\rho_{0}}\right)}^{\beta }-1\right]+p_{0}$$

where $\rho_{0}$ is the reference density, and $p_{0}$ is background pressure. β is a constant, usually β = 7; *B* is the pressure scale factor, usually set to $c^{2}\rho_{0}/\beta$.

The sound speed, *c*, in Equation (10) is estimated by the criteria shown below [42]:

(13) $$c^{2}=\max\left(\frac{V_{0}^{2}}{\delta },\frac{\nu V_{0}}{\delta L_{0}},\frac{FL_{0}}{\delta }\right)$$

where *V*_0_, *ν* and *L*_0_ are the velocity scale, kinematic viscosity and length scale, respectively. In order to meet the conditions for WCSPH fluids, the rate of change in fluid density should be less than 1%. At this point, $\delta =\Delta \rho /\rho_{0}$ should be set to 0.01.

The timestep dt in Equations (10) and (11) is a key factor that determines the calculation accuracy and efficiency, and it is typically controlled by three stability constraints: the CFL condition, mass force term and viscous diffusion term [43,44,45]. The complete criteria can be written as follows:

(14) $$dt=\min\;\left(0.25\frac{h_{i}}{c},0.25\min{\left(\frac{h_{i}}{f_{i}}\right)}^{1/2},0.125\frac{h^{2}}{\nu }\right)$$

where *h_i_* and ***f****_i_* are the smoothing length and acceleration of particle *i*, respectively.

### 2.2. Geometric Model and Simulation Settings

**Figure 2:**
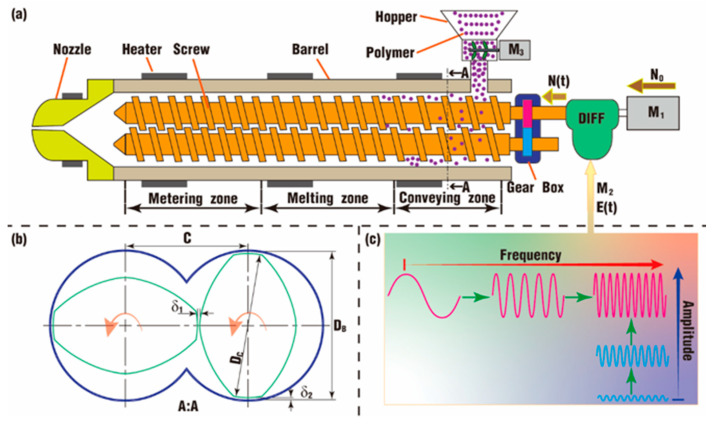
Differential pulsation system in TSEs (**a**): schematic diagram; (**b**) section view; (**c**) excitation samples.

As illustrated in Figure 2a, the twin-screw component can typically be divided into three zones: the solid conveying zone, the melting zone and the metering zone. The twin screws are coupled by a gearbox, and the tail end of one screw is linked to a differential (DIFF for short). The DIFF is driven by a major motor (M_1_ with a fixed speed, *N*_0_) and a control motor (M_2_ with a changing speed). M_2_ serves as a positive input excitation, denoted as *E*(*t*) = *A*sin (2*πft*), where *A* is the vibration amplitude, and *f* is the vibration frequency. The left-half shaft of the DIFF transfers the superimposition of the above two speeds (recorded as *N*(*t*), *N*(*t*) = *N*_0_ − *E*(*t*)) to the screw joined to the DIFF. With the *E*(*t*) changing sinusoidally, the *N*(*t*) changes accordingly, and the screws can thus obtain a sinusoidal pulsation speed. There is an additional motor for the hopper (M_3_) to keep the polymer materials in pace with the changing speed of the screws.

Figure 2b defines the key schemes for the model:

Outer diameter of the screws D_C_ = 60 mm.
Inner diameter of the barrel D_B_ = 62 mm.
Center distance between twin screws C = 51 mm.
Screw-screw gap δ_1_ = 1 mm.
Screw-barrel gap δ_2_ = 1 mm.

Figure 2c depicts some samples for the setting of various pulsation parameters in this study. Both the frequency and the amplitude can change alone or together. According to reference [35], the unit of the excitation, *E*(*t*), is degrees per second; the screw speed, *N*_0_, is assumed to be converted from revolutions per minute to degrees per second; and both the amplitude, *A*, and the frequency, *f*, should satisfy the following criteria:

(15) $$Af\leq 3N_{0}/\pi$$

Typically, six groups of frequencies and amplitudes are selected for simulation and detailed discussion; one is the steady state case, where the excitation amplitudes and frequencies are all 0, and the other five cases are under a dynamic state that is characterized by a non-zero excitation amplitude and frequency. In the five dynamic states, the frequency *f* is set as 1, 5 and 10 Hz in order, and the excitation amplitude *A* is set as 0.5, 2 and 6 degrees in order. Here, the influence of amplitude and frequency on the flow property is separately analyzed and discussed. In other words, the amplitude remains unchangeable at a fixed value of *A* = 6 degrees when the influence of excitation frequency is analyzed, and the frequency maintains an unchangeable value, *f* = 10 Hz, when the influence of excitation amplitude on the flow property is analyzed. Therefore, each case is a combination of amplitude and frequency and corresponds to one rotational speed. Here, a new term, vibration intensity (*VI*), is used to represent the vibration size, which is equal to the product of the amplitude and the frequency.

In Figure 2b, the least clearance occurs between the twin screws and the barrel, and it is set to be 1 mm in this study. According to the literature [46], there are at least four layers of particles required in the little clearances in order to guarantee calculation accuracy. Therefore, the distance between particles (denoted as *dp*) in this study is set to be 0.25 mm. As a result, there are 8,824,766 particles for the fluid and 1,165,876 particles for the boundaries. In SPH, the calculation time and used memory present exponential growth with the number of particles, and such a large number of particles will take months to complete the simulation for polymer fluid with a high viscosity [36,47,48,49]. Hence, the fluid in this study is assumed to be a Newtonian fluid with a very low value of 1 Pa·s for viscosity.

Other parameters that greatly affect the calculation time and memory are the sound speed and the timestep. Our past numerical experiments [35,36,38,43,48] for complex models like TSEs prove that a sound speed of 20 m/s is able to balance the viscous force so as to avoid particles penetrating the solid boundary. With the smooth length, *h*, typically set to 1.2 times that of *dp*, the time step, dt, can be calculated according to Equation (14), *dt* = 3.75 × 10^−6^ s.

Based on the above discussion, the analysis parameter setting for the six cases can be arranged and obtained in ascending order according to the *VI* value, as shown in Table 1.

## 3. Results and Discussion

### 3.1. Velocity and Pressure from an Overall Perspective

**Figure 3:**
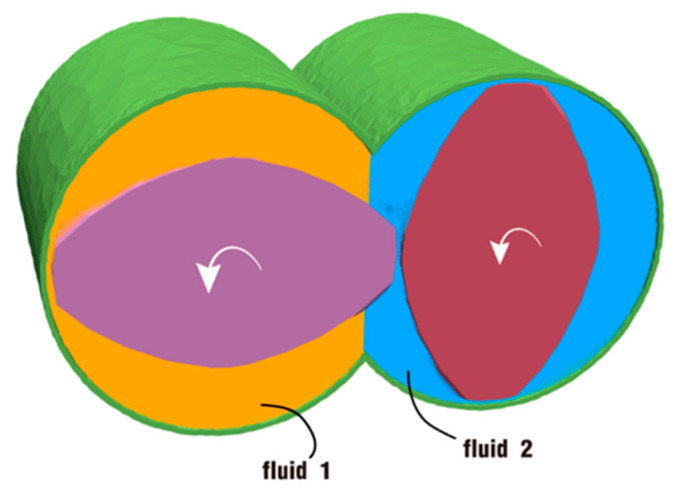
Initial position setup for fluid 1 and fluid 2 in simulated cases.

As shown in Figure 3, the channel between the barrel and the twin screws is in a fully filled state and usually split into two geometrically equal parts. The left part of the channel (marked in orange) is filled with fluid 1, and the right part (marked in blue) is filled with fluid 2, which represent two different materials for mixing. As indicated by the arrows in the picture, the twin screws rotate counter-clockwise at the same speed, and this speed differs as a result of the different excitation for each simulated case. The two fluids completely cover the space between the barrel and the screws and are dragged forward in the channel by the rotating screws.

#### 3.1.1. Velocity Distribution

**Figure 4:**
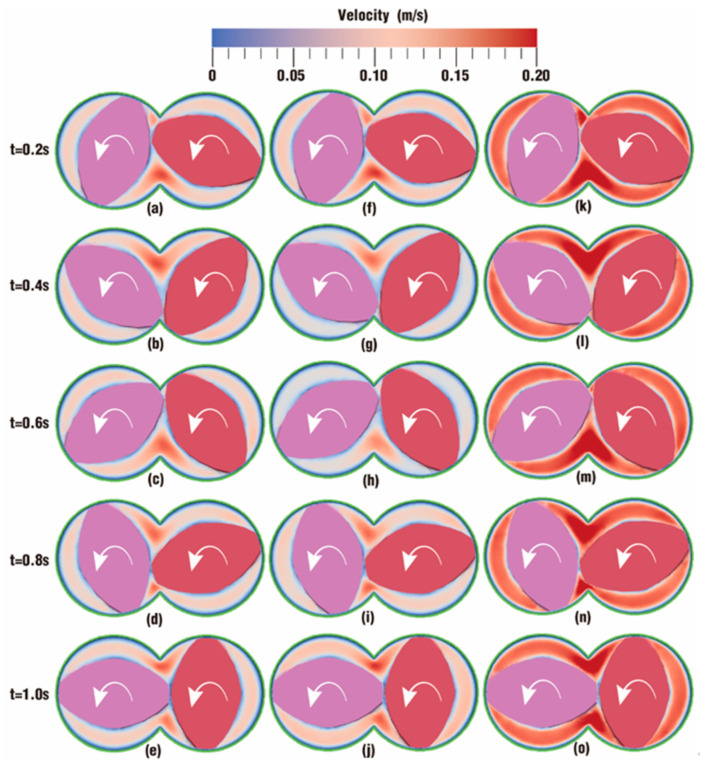
Effect of frequencies on velocity distribution at certain timesteps: case 0 (**a**–**e**), case 1 (**f**–**j**), case 2 (**k**–**o**).

**Figure 5:**
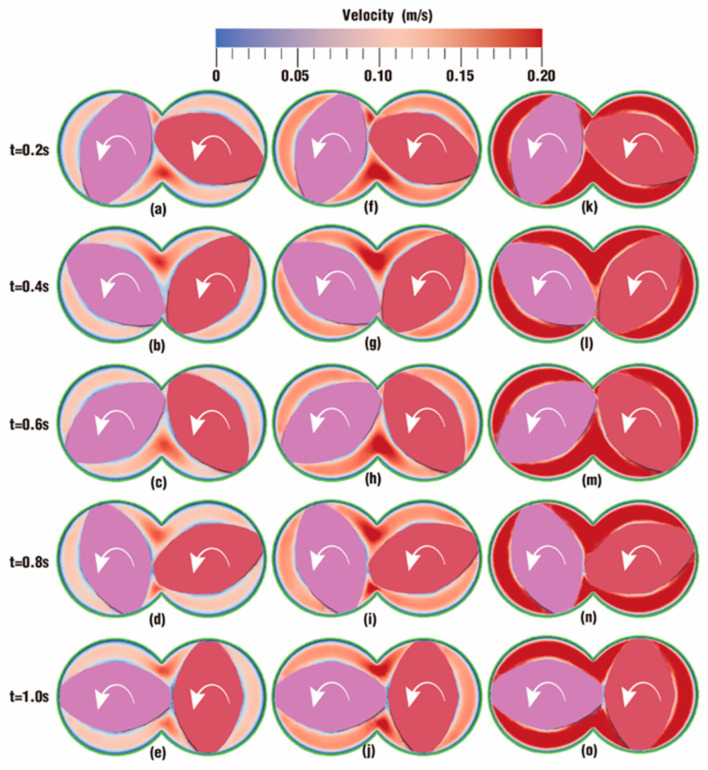
Effect of amplitudes on velocity distribution at certain timesteps: case 1 (**a**–**e**), case 3 (**f**–**j**) and case 5 (**k**–**o**).

Figure 4 and Figure 5 describe the comparison contours of velocity distribution at certain timesteps over one rotation between the six cases set in Table 1. The colors in these contours represent different velocity values. Since the velocity values of the six cases are in a large span, the color in most cases becomes darker if the range of the color scale is too small, and in this situation, it is not easy to distinguish the cases with larger values. If the value of the color scale is too large, the color in most cases becomes too light to be clearly identified. After several attempts, cases can be clearly distinguished when the value range of the color scale is determined between 0 m/s (denoted as blue) and 0.2 m/s (denoted as red).

As depicted in Figure 4, the contours of vibration distribution in the flow channel are compared under the action of different frequencies at several timesteps. It can be seen from the figure that there is much similarity between case 0, case 1 and case 2. Contours in the intermeshing zone take on a redder color than those in the rest zone of the channel, indicating that velocities in the intermeshing zone are larger than those in the non-meshing zone. This is mainly because the twin screws alternately pass through the meshing zone and form a strong shear and dragging action. The color is much lighter along the outer surface of the twin screws and the inner surface of the barrel due to wall adhesion, indicating a rather lower value of velocities in these areas.

By contrast, the contours in case 0 and case 2 are almost the same, suggesting that the velocities in both cases evolve similarly. Typically, at the same timesteps. *t* = 0.2 and 0.8 s, there is hardly a difference in contours between Figure 4a and Figure 4f or between Figure 4d and Figure 4i. At timesteps *t* = 0.4 and 0.6 s, the contours in case 0 are a bit deeper than those in case 2, while at *t* = 1.0 s, the contour in case 0 is a little lighter than that in case 2. However, when the frequency increases from 1 Hz in case 2 to 5 Hz in case 4 at the same amplitude, *A* = 6 degrees, it can be obviously observed that the contours become significantly redder, which reveals that particles in case 4 have a much greater velocity. In addition, from the parameter settings for case 0, case 2 and case 4 in Table 1, it can be inferred that a low frequency for the superimposed excitation has little impact on the velocity distribution, while this impact becomes much more important when the frequency increases.

The contours of vibration distribution in the flow channel are also compared under the action of different amplitudes with the same frequency (*f* = 10 Hz) at several timesteps, as shown in Figure 5. The overall trend in Figure 5 is similar to that in Figure 4. Obviously, the larger the amplitude is, the redder the contour becomes. Therefore, particles would experience a greater velocity when the excitation frequency is set to a larger value. Note that the *VI* values of case 0, case 1 and case 2 are 0, 5 and 6 degrees per second respectively, and the contours for those three cases are nearly the same. When the *VI* value increases by up to 20 degrees per second for case 3 and 30 degrees per second for case 4, the contours become a little redder. When the *VI* value increases by up to 60 degrees per second for case 5, the contour becomes the reddest in nearly all channels. These results imply that the enhancement of the superimposed vibration field on the velocity field will become more apparent as the *VI* value increases.

**Figure 6:**
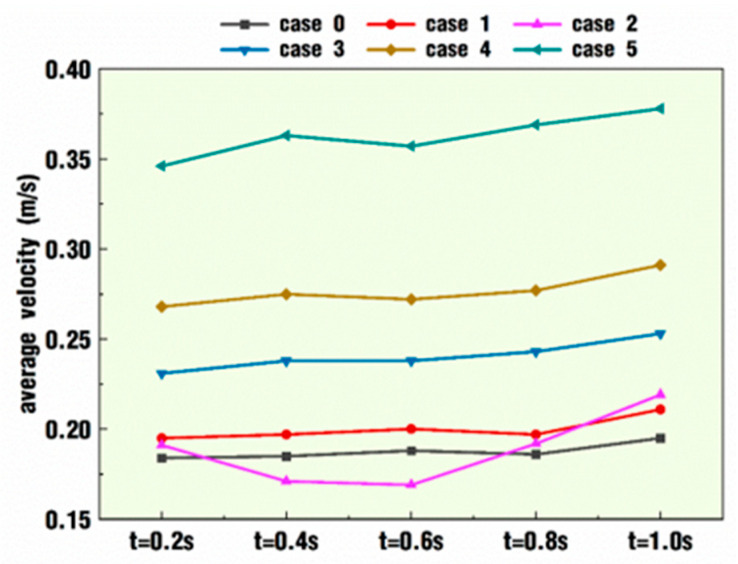
Average velocity of selected timesteps corresponding to Figure 4 and Figure 5.

Following the above contours of velocity distribution, Figure 6 quantitatively describes the averaged velocity at each timestep by collecting the top 1000 velocity values for the six cases. Except for case 2, it is quite apparent that cases under pulsation excitation conditions have greater velocities than the normal condition, case 0. Moreover, the difference is gradually enlarged as the *VI* value increases. Specifically, the average velocity of case 5 at each timestep is almost twice as much as that of case 0. However, case 2 shows a different change against this trend. At timesteps *t* = 0.2 and 0.8 s, the average velocity of case 2 is slightly greater than that in case 0; at timesteps *t* = 0.4 and 0.6 s, the situation is just the opposite, and the former is smaller than the latter; at timestep *t* = 1 s, the former is far beyond the latter. The above results show that the superposition of pulsation excitation, as a whole, could improve the average velocity in the flow channel at some timesteps, and there are some combination pairs of amplitude and frequency that would lower the average velocity.

The above conclusions are analyzed and obtained at several timesteps, which give information regarding the velocity in the flow channel at certain moments, and the following content will focus on the analysis of the max velocity and other parameters in the whole simulation period. Due to the huge number of particles in this simulation, and in order to avoid the difficulty of analysis caused by the violent oscillation of the data, the maximum parameter of each moment is smoothed, especially for the velocity and pressure. The smoothed method is as follows: at each selected timestep, the average of the top 20 values for one parameter is computed and chosen as the maximum value of the above special parameter at that timestep.

**Figure 7:**
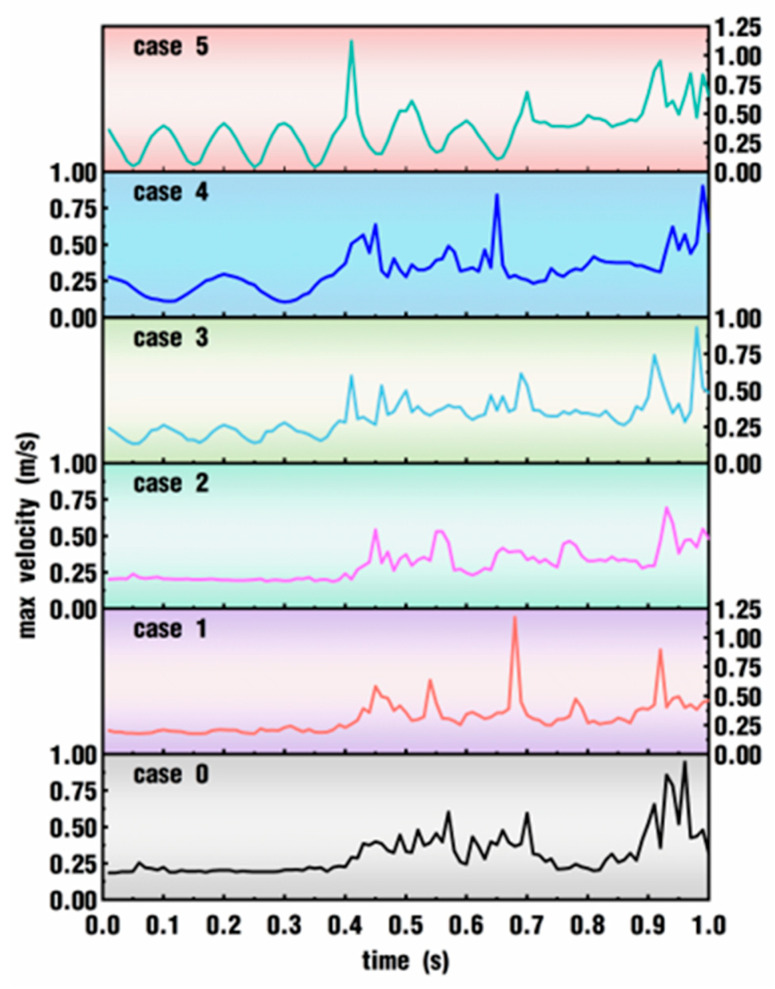
Effect of excitation parameters on max velocity within the whole simulation time.

Figure 7 depicts the effect of excitation parameters on max velocity within the whole simulation time for the six cases. It can be seen from the figure that the max velocities for case 0, case 1 and case 2 are stable within the beginning 0.4 s, while the max velocities of the three cases take on a sharp fluctuation from 0.4 s to the end of the simulation time. In the remaining three cases, the fluctuation starts periodically right from the beginning of the simulation, which is much earlier than in the first three cases. Moreover, the amplitude and frequency values of velocity fluctuation are to some extent consistent with the amplitude and frequency set for the pulsation excitation of those cases. For example, case 3 and case 5 have a frequency of 10 Hz, and case 4 has a frequency of 5 Hz. Accordingly, within the first 0.4 s, the fluctuation numbers of the max velocity in the former two cases are nearly twice as many as those in the latter case. It can also be seen from the figure that, except for the periodical fluctuation within the first 0.4 s, the large fluctuation develops earlier and earlier as the value of *VI* increases.

**Figure 8:**
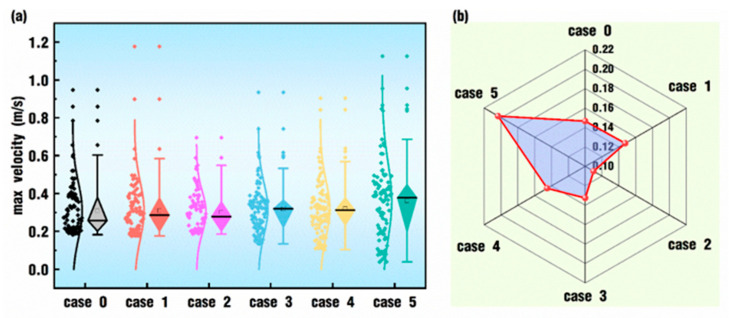
Statistical results of max velocity corresponding to Figure 7: (**a**) data distribution; (**b**) standard deviation.

The comparison of the statistical results of max velocity corresponding to Figure 7 is presented in Figure 8. It can be seen from Figure 8a that case 0, case 1 and case 2 have shorter box bodies and lower shadow lines, indicating that their maximum velocity values are relatively concentrated. In the latter three cases, the maximum velocity values are increasingly dispersed. At the same time, the median of the maximum velocity also increases gradually with the addition of the *VI* value. On the whole, the fluctuation range of the maximum and minimum values decreases first from case 0 to case 2 and then increases gradually from case 3 to case 5. The fluctuation of the six cases can be better seen in Figure 8b. Obviously, case 2 has the minimum standard deviation, and case 5 has the maximum standard deviation. Combining Figure 7 and Figure 8, the average velocity in the whole flow field enlarges with the increase in *VI* values set in the superimposed pulsation excitation, and the range of velocity fluctuation also expands gradually.

#### 3.1.2. Pressure Distribution

**Figure 9:**
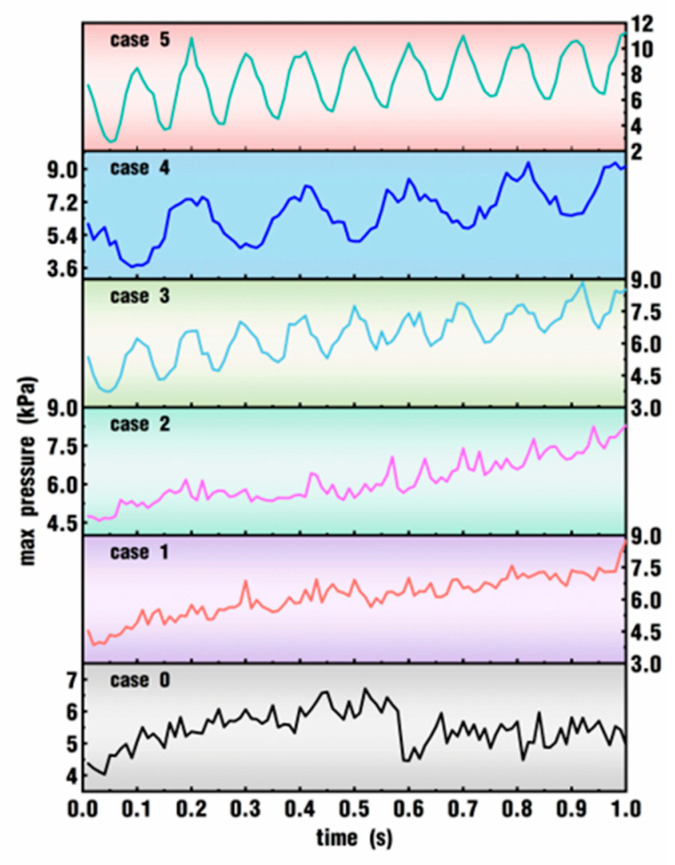
Effect of excitation parameters on max pressure within the whole simulation time.

Figure 9 analyzes the effect of excitation parameters on max pressure within the whole simulation time for the six cases. In the figure, the evolution of max pressure as a function of time is quite different from that of max velocity in Figure 7. The curves of max pressure in Figure 9 have much smaller oscillation amplitudes but slightly greater frequencies than those in Figure 7. In case 0, the max pressure goes up steadily within the first 0.5 s and then oscillates horizontally until the simulation ends. When a small *VI* value is applied, such as 5°/s for case 1 and 6°/s for case 2, the maximum pressure in the flow channel oscillates with a slight amplitude during the whole simulation time. When a larger *VI* value is used, typically 20°/s for case 3 and 60°/s for case 5, the maximum pressure takes on an apparent upward and sinusoidal oscillation with a much larger amplitude and frequency. Furthermore, case 4 and case 5 have the same vibration amplitude, while the frequency of the latter is twice as large as that of the former. Coincidentally, the curve of max pressure in case 5 almost has the same oscillation amplitude as that in case 4, and the oscillation frequency of the curve in case 5 is twice that in case 4. In other words, the oscillation frequency complies with the original frequency set for the speed excitation on the twin screws, as listed in Table 1.

**Figure 10:**
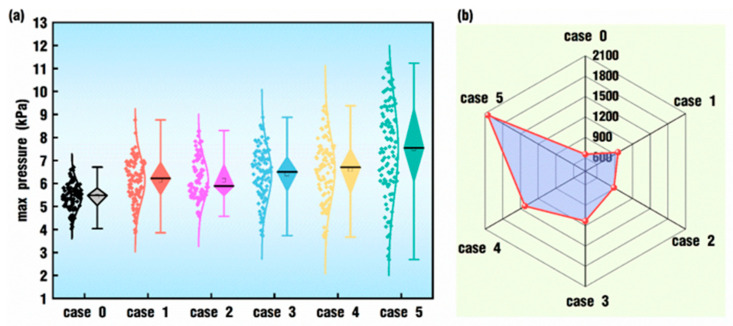
Statistical results for max pressure corresponding to Figure 9: (**a**) data distribution; (**b**) standard deviation.

A comparison of the statistical results of max pressure corresponding to Figure 9 is shown in Figure 10. In Figure 10a, case 0 has the shortest box bodies and shadow lines, and it also has the lowest median and upper whiskers, indicating that its pressure values are the most concentrated. As the value of *VI* increases from case 1 to case 5, there is a trend where the median becomes larger and larger, the range between the lower whisker and the upper whisker grows longer and longer, and the data distribution fragments increase. In Figure 10b, there is a better comparison of the standard deviation among the six cases. Case 0 has the minimum standard deviation, and case 5 has the maximum standard deviation. Combining Figure 8 and Figure 10, the results indicate that there are similarities and differences between the effects of excitation parameters on both the max velocity and the max pressure in the whole flow field. On the one hand, the superposition of pulsation excitation on the twin screws could indeed enhance the velocity field and the pressure field, and the enhancement could be strengthened with the increase in the *VI* value. On the other hand, periodical oscillation happens apparently only for some *VI* values and in some intervals for the effect of excitation on the max velocity, while there are obvious sinusoidal oscillation periods right from the beginning of the simulation for cases with large enough *VI* values.

**Figure 11:**
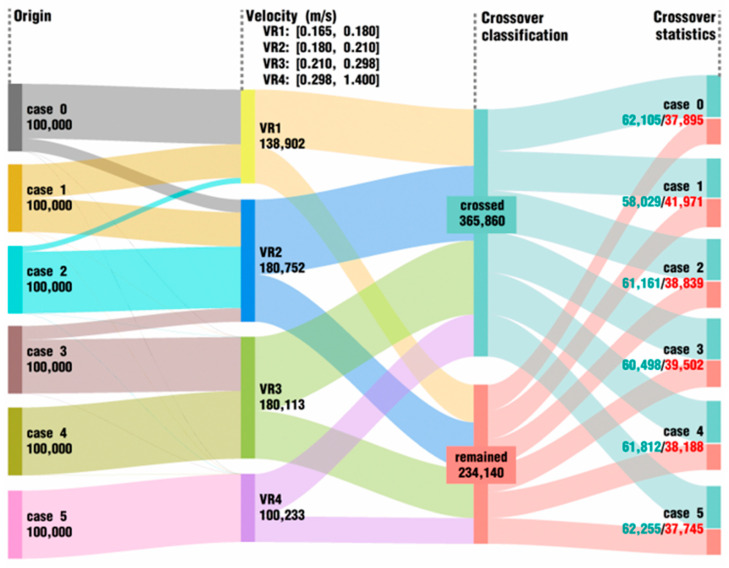
Data direction of top 100,000 particles in velocity at last timestep for each case.

**Figure 12:**
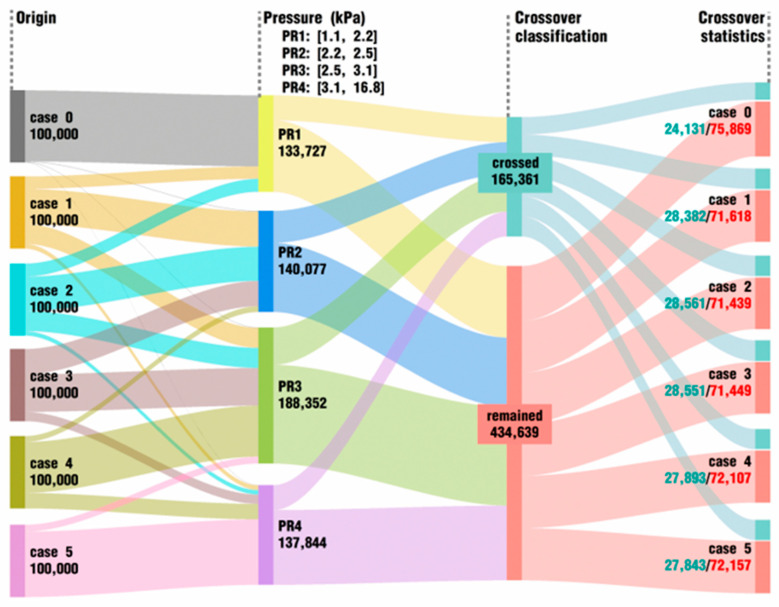
Data direction of top 100,000 particles in pressure at last timestep for each case.

In order to more clearly show the velocity distribution, pressure distribution, and crossover flow of corresponding particles in the entire channel, the top 100,000 particles in each case are selected for statistics based on the velocity and pressure, respectively, as shown in Figure 11 and Figure 12. Either in the analysis of the velocity index (VR for short) or in the analysis of the pressure index (PR for short), the overall range is unevenly divided into four intervals, which are used to describe all values in the six cases as well as possible. In Figure 11, according to the velocity values, 100,000 top particles are acquired for each case; then, these particles are sorted from the smallest velocity interval to the greatest velocity interval, and finally, the particles are once more classified and counted based on whether one particle crosses from its origin zone into the other zone. It can be clearly seen that most of the particles in case 0 lie in the interval between 0.165 and 0.180 m/s, and there are very few particles that have larger velocity values than 0.180 m/s. By contrast, particles in those cases under vibration excitation conditions have a relatively large velocity, and these velocity values gradually get bigger and bigger as the *VI* value for the excitation parameters increases. For example, all the velocity values of particles in case 5 are greater than 0.298 m/s and are nearly twice as large as those in case 0. As a whole, about 61% of the 600,000 particles can cross into the opposite fluid region. However, as listed in the crossover statistics, there is no obvious relationship between the number of crossover particles and the *VI* values. Compared with case 0, the number of crossed particles increases in some cases, while in other cases, the number decreases.

Figure 12 covers the data direction of 100,000 particles selected by the pressure index in each case at the end of the simulation. From the diagram, it can be easily observed that particles in case 0 are almost in a low range of pressure between 1.1 and 2.2 kPa, and only a few particles have larger values beyond that range. In cases 1–4, more and more particles suffer from a greater pressure field, and in case 5, the majority of particles have pressure values larger than 3.1 kPa, which are nearly three or more times as big as those in case 0. To be more precise, compared with the normal condition, case 0, cases under superposed excitation conditions can greatly improve the pressure in the flow field. In short, similar to the effect of excitation parameters on the velocity in Figure 11, the *VI* values of vibration excitation play an important positive role in increasing the pressure in the flow channel.

As for the crossover particles, there are distinct differences between Figure 11 and Figure 12. Here, the crossover particles take up about 28% of the original 600,000 particles, while the proportion for the latter is about 61%, and the total number of crossover particles has been reduced by more than half. This indicates that a higher velocity is more conducive to particle crossing than a higher pressure. In addition, the number of crossover particles in the cases with excitation is approximately 4000 more than that in case 0, which has no excitation. Nevertheless, adding *VI* values hardly has any enhancing effect on improving the number of crossover particles among the cases under vibrating excitation conditions. In fact, case 4 and case 5 have larger *VI* values than cases 1, 2 and 3, but the former two cases both obtain a slightly smaller number of crossover particles than the latter three cases do.

From the above results and discussion, and from an overall view of the fluid particles, it can be concluded that, on the one hand, the superposed rotation excitation on the twin screws could indeed allow the particles to acquire higher, more discrete velocity and pressure values, and these values increase with the addition of *VI* values set for excitation. On the other hand, for all cases, greater velocity values could lead to a greater proportion of crossover particles than the pressure values do, but the enhancement of excitation parameters, which can improve flow physical properties such as velocity or pressure values, does not make a distinct difference in the number of crossover particles, even though there is a little increase in the crossover number in the pressure situation.

### 3.2. Particle Distribution from Slice Views

**Figure 13:**
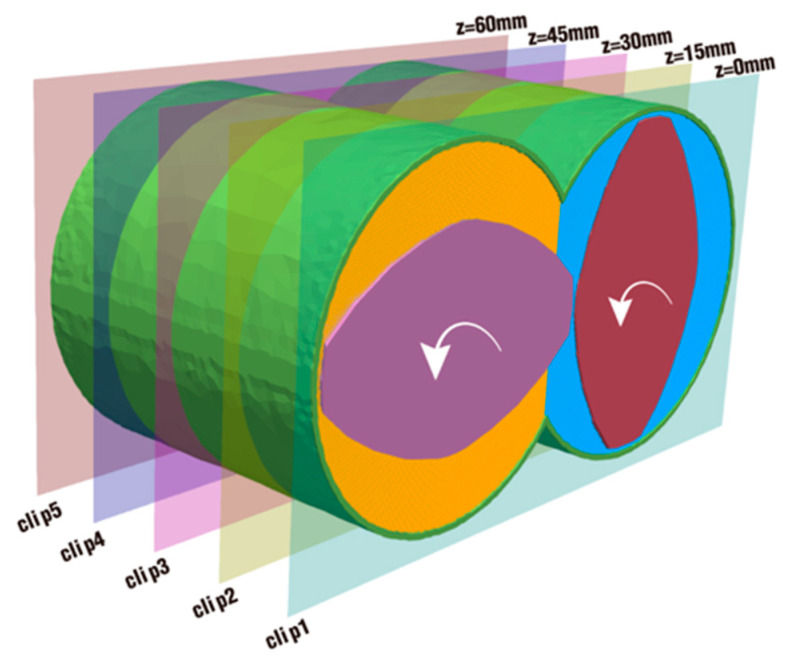
Five clips established perpendicular to the *Z*-axis.

In Section 3.1, the overall velocity and pressure are depicted and discussed in detail from a maximum and statistical point of view, but they are a bit abstract, especially regarding the crossover statistics, and this section provides some concrete visual perspectives to make up for the above deficiencies. To visualize the flow situation inside the channel for the twin-screw component, this section employs five clips with equal spacing, 15 mm from the inlet to the outlet, specifically along the *Z*-axis of the flow channel, as displayed in Figure 13. For the sake of easy description, the first slice with *z* = 0 mm is clip 1, the second slice with *z* = 15 mm is clip 2, and so on. The following content will mostly refer to the effect of excitation parameters set in Table 1 on particle distribution from the view of these clips.

**Figure 14:**
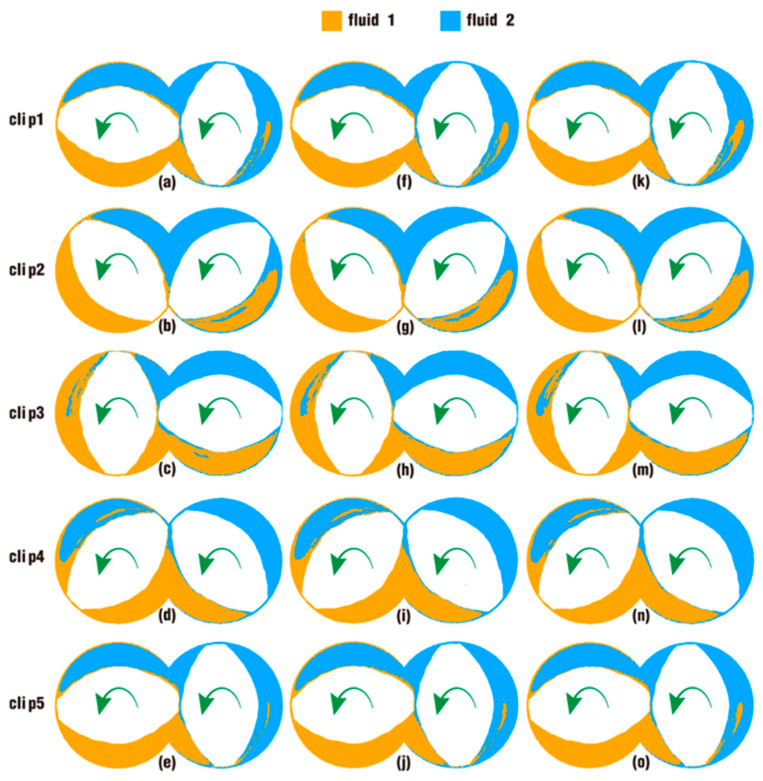
Effect of frequencies on particle distribution in clips after one rotation: case 0 (**a**–**e**), case 2 (**f**–**j**) and case 4 (**k**–**o**).

Figure 14 displays some snapshots to describe the effect of excitation frequencies on the particle distribution of clips at the end of the simulation. Since the model used in DualSPHysics adopts the STL format, the twin screws become curves instead of planes when the slices are established; thus, the twin-screw models are omitted here, and the shape of the twin screws is represented by blank areas. Obviously, the snapshots clearly show the distribution of particles at a specific place and moment, which provides an intuitive perspective of the mixing distribution for particles in fluid 1 and fluid 2. On the whole, with the superposition of vibration excitation and the increase in vibration frequency, it is easy to identify the difference in the mixing distribution of two fluid particles in some cases. In clips 1 and 5, in the upper left regions of Figure 14a,e,f,j,k,o, where they are originally set for fluid 1, the areas are nearly taken up by fluid 2. In clip 3, in the lower right regions of Figure 14c,h,m, where they are initially the areas for fluid 2, now, the places are mostly occupied by particles of fluid 1.

In some local areas, only slight differences can be found between cases. Specifically, in the lower right regions of Figure 14a,f,k on clip 1 and Figure 14e,j,o on clip 5, the original areas for fluid 2, there are more particles of fluid 1 in Figure 14f,k than in Figure 14a. Similarly, in the upper left regions of Figure 14c,h,m on clip 3, the original areas for fluid 1, there are more particles of fluid 2 in Figure 14h than in Figure 14c, and in Figure 14m, there are more particles of fluid 2 than in Figure 14h. By contrast, the contours of particle distribution in the remaining places are almost identical. These results show that the excitation frequencies could have a conducive effect on particle distribution, while the difference in that effect between cases is not apparent enough to be clearly distinguished from the contours.

**Figure 15:**
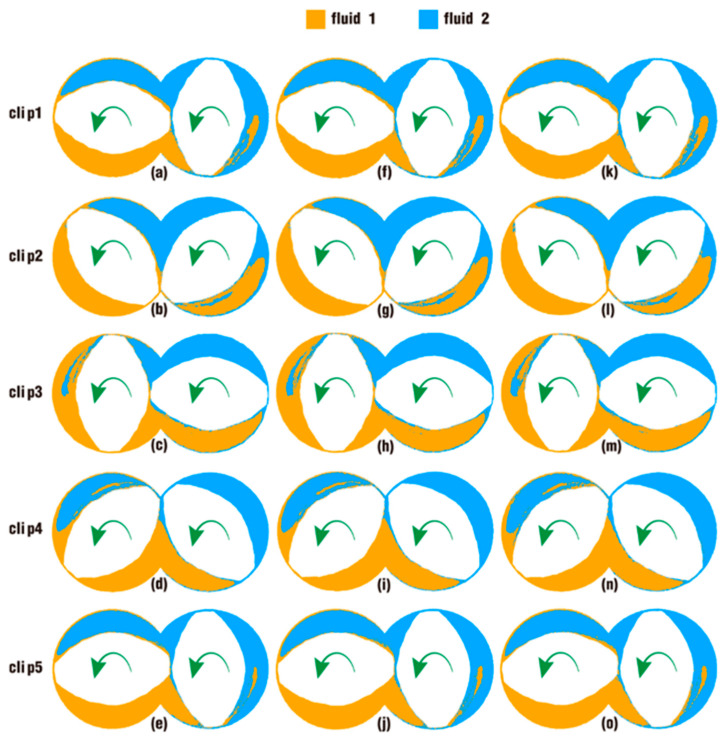
Effect of amplitudes on particle distribution in clips after one rotation: case 1 (**a**–**e**), case 3 (**f**–**j**) and case 5 (**k**–**o**).

Following the above analysis, Figure 15 lists some snapshots to illustrate the effect of excitation amplitudes with a fixed frequency (*f* = 10 Hz) on particle distribution on clips after one rotation of the twin screws. Similar to the results in Figure 14, except for the apparent distinction in clips 1 and 5, only slight differences can be found in some local areas between the three cases. Generally speaking, in the lower right region of Figure 15a,f,k on clip 1, Figure 15b,g,l on clip 2, and Figure 15e,j,o on clip 5, the original areas for fluid 2, there are more particles of fluid 1 in the last two contours than in the first contour on each clip. Likewise, in the upper left region of Figure 15c,h,m on clip 3, the originally area for fluid 1, there are more particles of fluid 2 in Figure 15h,m than in Figure 15c. In the remaining clip, clip 4, the three contours of particle distribution are almost indistinguishable. Similarly, the above results show that the excitation amplitudes with the same frequency are beneficial for particle distribution to some extent, whereas this effect could not be obviously seen between cases just from the contours alone.

**Figure 16:**
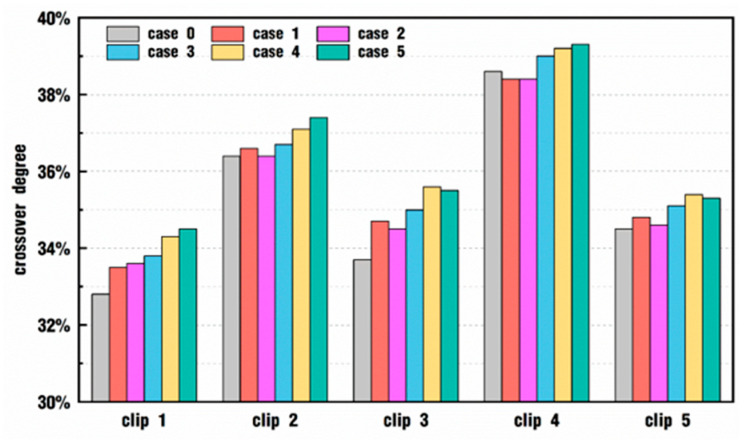
Comparison of crossover degrees of particles on each clip.

**Figure 17:**
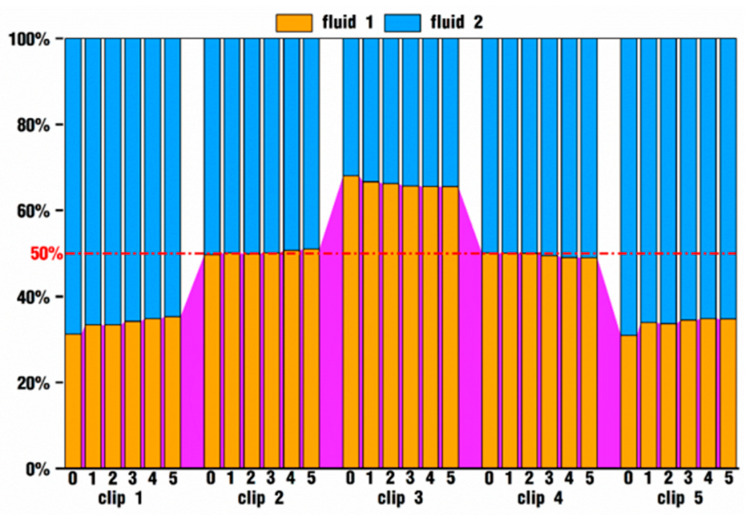
Ratio of crossover particles from fluid 1 versus those from fluid 2.

In order to quantitatively analyze the influence of vibration parameters on particle distribution, the particles on each clip corresponding to Figure 14 and Figure 15 are statistically classified, and the proportion of the crossover particles to all the particles on each slice is computed and listed, as shown in Figure 16. In addition, the crossover particles on each clip are further subdivided to confirm whether one particle is from fluid 1 or from fluid 2 and to count the proportion of those particles, as drawn in Figure 17.

In Figure 16, it is evident that, at the same time, the crossover degrees of particles at different positions in the flow channel are also different. For one thing, the crossover degree at the entry position, clip 1, is the smallest under various velocity conditions, followed by a slight increase at clips 3 and 5 and a large improvement at clips 2 and 4. Since the particles have to pass through the location of each clip from the entrance to the exit, the number of crossover particles in different places is constantly changing, which indicates that some of the crossover particles have backflow into their original fluid area. Furthermore, the crossover degree apparently increases with the addition of *VI* values to clip 1 and clip 3, while in other clips, the crossover degree of case 2 is basically equal to or slightly bigger than that of case 0. This result suggests that high enough *VI* values could strengthen the crossover degree, thus providing the particles with a higher proportion of mixing processes.

Figure 17 counts the proportion of crossover particles from fluid 1 versus fluid 2 in each clip among the simulated cases corresponding to Figure 16. In clip 1 and clip 5, specifically, at the inlet and the outlet parts of the flow channel, the proportion of crossover particles from fluid 1 is about from 30% to 35%, and accordingly, the proportion of crossover particles from fluid 2 is about from 70% to 65%. In clips 2 and 4, the proportion of crossover particles from the two fluids is almost equal, accounting for about 50%. In clip 3, the proportions are about 65% and 35% for fluid 1 and fluid 2, respectively. These results are in exact agreement with those from Figure 14 and Figure 15, where in clips 1 and 5, there are more particles from fluid 2 in the upper right regions originally set for fluid 1, and in clip 3, there are more particles from fluid 1 in the lower right regions originally set for fluid 2. However, as the *VI* value increases, the proportion does not make any obvious difference, which indicates that the excitation parameters have an almost negligible effect on the proportion of crossover particles from two fluids of the same clip.

### 3.3. Tracking Particles from a Local View

**Figure 18:**
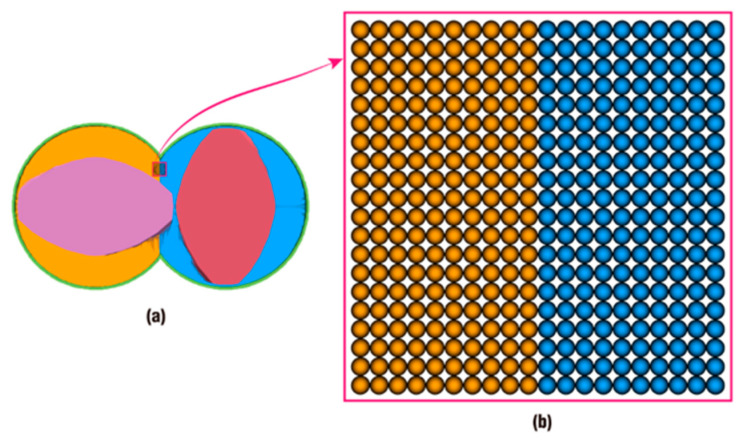
Initial settings for tracking particles: (**a**) location of tracking particles; (**b**) the amplified graph.

Next, the influence of vibration parameters on the flow field is studied by tracking tracer particles in the fluid region. The position of tracking particles is located in the meshing zone of the twin screws. In each case, 200 particles are selected for fluid 1 and 200 for fluid 2, and thus, a total of 400 particles are collected, as shown in Figure 18. The tracking particles are set at the beginning of the simulation, and the new positions, velocity and pressure of the particles are gathered and counted at the end of the simulation.

**Figure 19:**
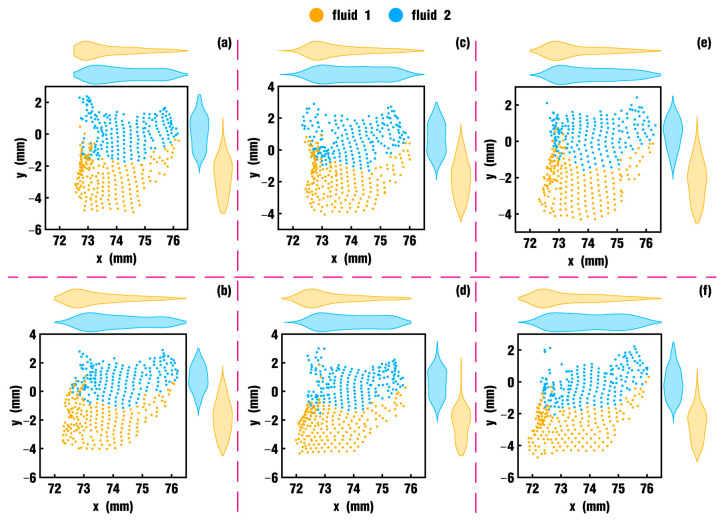
Position distribution of tracking particles for each case at the end of the simulation: (**a**) case 0; (**b**) case 1; (**c**) case 2; (**d**) case 3; (**e**) case 4; (**f**) case 5.

Here, the position distribution of the 400 particles for each case at the end of the simulation is tracked and drawn, as illustrated in Figure 19. The figure reveals that particles of case 0 lie mainly within a range of [72.8, 76] mm in the horizontal coordinate axis, and particle agglomeration occurs in many places. The particle distribution of case 1 moves to the left, while the right position is basically unchanged, and in case 5, the particles are located between 71.8 and 76 mm in the *X* direction with a more uniform distribution and less agglomeration. Therefore, with the increase in the *VI* value, the distribution of particles is more dispersed, and the overall movement to the left for the particles is more obvious. Next, the physical information of the tracking particles in the above cases is statistically analyzed to obtain quantitative comparison results. Here, the velocity and pressure information of the tracking particles in each case is analyzed.

**Figure 20:**
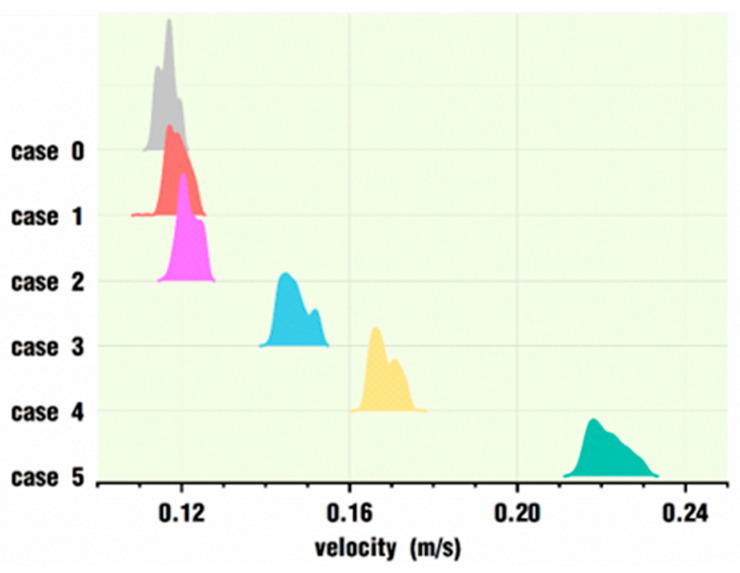
Velocity distribution of tracking particles for each case at the end of the simulation.

Figure 20 depicts the velocity distribution of the tracking particles for the six cases at the end of the simulation. Each curve corresponds to the velocity distribution of 400 particles in a case. It is clear that all the distribution curves are relatively concentrated, and the overall distribution curve for each case gradually moves to the right of the horizontal coordinate axis with the increase in *VI* values. Moreover, as the *VI* value increases, the spacing between the curves becomes larger and larger. That is, the velocity of the corresponding case increases more. In other words, a small *VI* value set for one case would not improve the velocity of the particles in a case too much compared with the normal rotation condition, case 0, while a large enough *VI* value would allow the particles to experience a much greater velocity compared with case 0.

**Figure 21:**
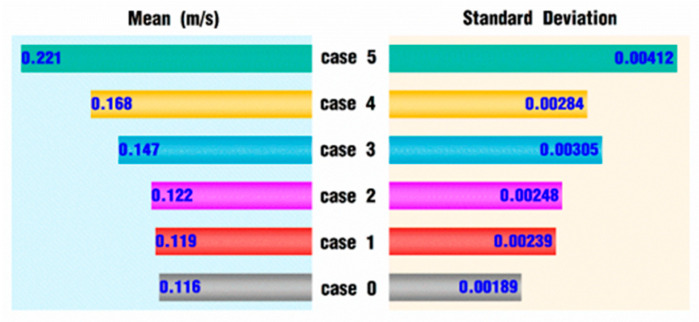
Velocity statistics for tracking particles at the end of the simulation.

Figure 21 presents the mean and standard deviation of the velocity values of the 400 tracking particles in each case under different pulsating excitation parameters corresponding to Figure 20. As can be seen from the figure, the average value of 0.116 m/s in case 0’s working state is the minimum for all the cases, and the average velocity value of the tracking particles increases to varying degrees for the other rotational speeds, which are superimposed by the pulsating excitation. The average velocity increases slightly with smaller *VI* values and rapidly with larger *VI* values. The average velocity is 0.221 m/s in case 5, which is about twice as large as that of case 0.

It can also be seen from Figure 21 that the standard deviation of the velocity varies in almost the same way as the mean value, and the standard deviation under the state of case 0 is the smallest, with a value of 0.00189, which indicates the smallest fluctuation of velocity values for the 400 tracking particles. The standard deviation value of case 5 is 0.00412, which indicates the largest fluctuation of velocity values for the 400 tracking particles. In general, the superimposed pulsation excitation can greatly improve the velocity values for the tracking particles, while it also increases the fluctuation of the velocity values. These results are quite consistent with the above results discussed in Section 3.1.

**Figure 22:**
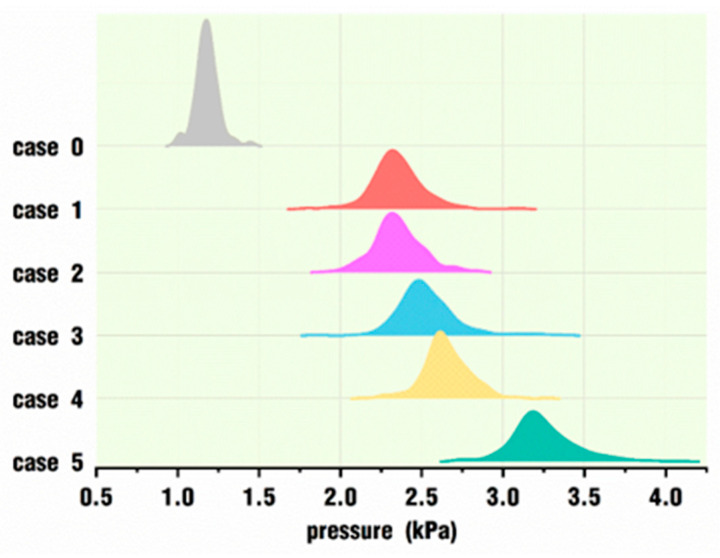
Pressure distribution of tracking particles for each case at the end of the simulation.

Figure 22 shows the pressure distribution of the tracking particles for the six cases at the end of the simulation. Similar to Figure 20, each curve also corresponds to the pressure distribution of 400 particles in a case. It can be clearly seen that all of the distribution curves are relatively concentrated, and the overall distribution curve for each case gradually moves to the right of the horizontal coordinate axis with the increase in *VI* values. Different from Figure 20, all the curves of cases under superposed pulsation excitation are far away from case 0, and there are smaller differences from case 1 to case 5. Moreover, the distribution ranges for cases 1–5 are much larger than those in Figure 20. Therefore, once the pulsation excitation is loaded on the twin screws, the pressure is increased by nearly two times that of case 0, and the pressure value improves slightly as the *VI* value increases.

**Figure 23:**
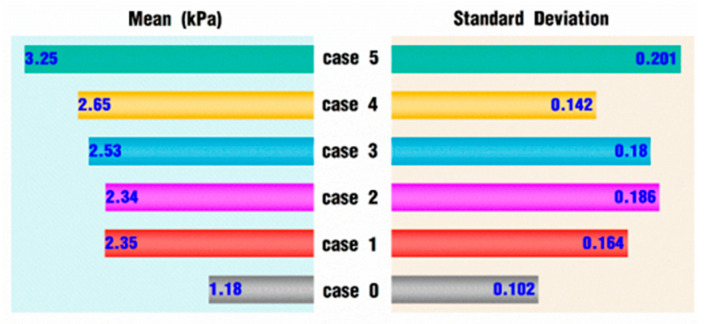
Pressure statistics for tracking particles at the end of the simulation.

Finally, Figure 23 compares the mean and standard deviations of the pressure values of the 400 tracking particles in each case under different pulsating excitation parameters, corresponding to Figure 22. Compared with case 0, without pulsating excitation, the mean and standard deviations of the pressure values with the superposition of the pulsating excitation on the rotational speed do not monotonically increase or decrease with the change in *VI* values, showing some very random characteristics. The average pressure in case 0 is 1.18 kPa, and the smallest average pressure under pulsation excitation is 2.34 kPa in case 2. The latter is almost twice as large as the former. On the whole, the average pressure increases slightly under cases 1–5 with the increase in *VI* values. The standard deviation of the pressure varies in a different way. The standard deviation of case 0 is the smallest, with a value of 0.102, which indicates the smallest fluctuation in velocity values for the 400 tracking particles. In cases with pulsation excitation, the standard deviation first increases to 0.186 in case 2, then drops down to 0.142 in case 4, and finally reaches the maximum 0.201 in case 5, which indicates the largest fluctuation in pressure values for the 400 tracking particles.

Given the above results and discussion on the distribution of position, velocity and pressure for the 400 tracking particles in each case, it can be seen that the superimposed pulsation excitation can apparently improve the physical properties of the tracking particles. With the increase in *VI* values, the physical properties become greater and greater, while the values fluctuate more and more sharply. These results, as discussed in the above sections, suggest that a larger *VI* value can enhance the variation range for physical parameters and thus allows the particles in the channel to acquire a relatively greater flow parameter. According to the literature [26], the total crystallinity is 42% for traditional injection molded samples, and this value increases to 52%, 59% and 66% for examples fabricated by the 1-Hz, 4-Hz and 30-Hz vibration-assisted processes, respectively. It can be seen that the crystallinity improves under the vibration excitation relative to the traditional condition, and the value of crystallinity increases as the vibration frequency increases. This trend is basically in agreement with the results obtained in this research.

### 3.4. Expanding Amplitudes and Frequencies

The results in the above sections focus on field parameters such as velocity, pressure and particle distribution through qualitative analysis of contours and statistical quantitative analysis from the perspectives of the overall channel, the *Z*-axis clips, and the tracking particles in the meshing zone. The influence of several specific vibration amplitudes and frequencies on the above field parameters is compared with the normal condition, case 0. It is worth noting that the amplitude and frequency in the superimposed vibration field have several discrete values, and the analysis conclusion only reflects part of the information on this influence. It is necessary to generally expand these amplitudes and frequencies in order to obtain a relatively good conclusion. Therefore, more amplitude and frequency values are studied in this section. The amplitudes are set as 0.5, 1, 2, 4, and 6 degrees, and the frequencies are 1, 2, 5, 8, and 10 Hz, respectively. The two parameters are independent and orthogonally combined; thus, there are 25 cases, which constitute the basic analysis cases.

**Figure 24:**
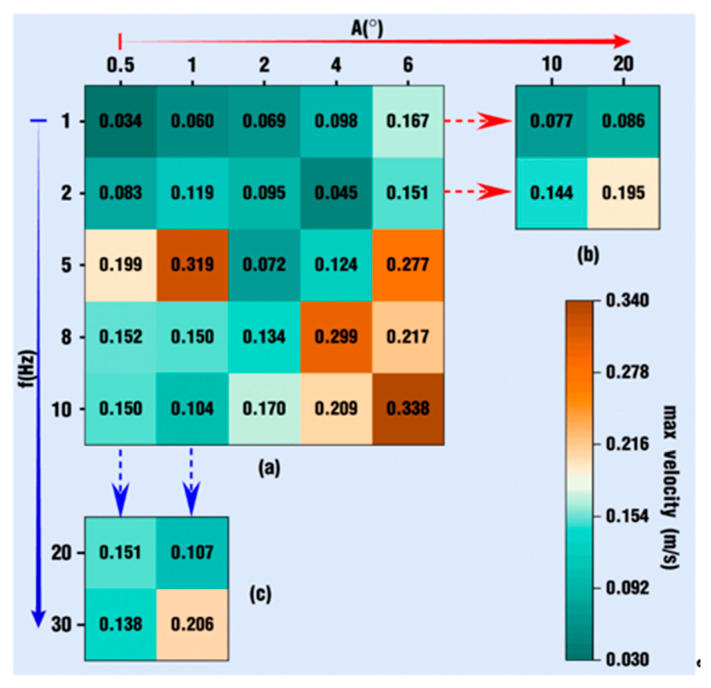
Max velocity of expanding cases: (**a**) normal conditions, (**b**) high amplitude and low frequency, and (**c**) low amplitude and high frequency.

**Figure 25:**
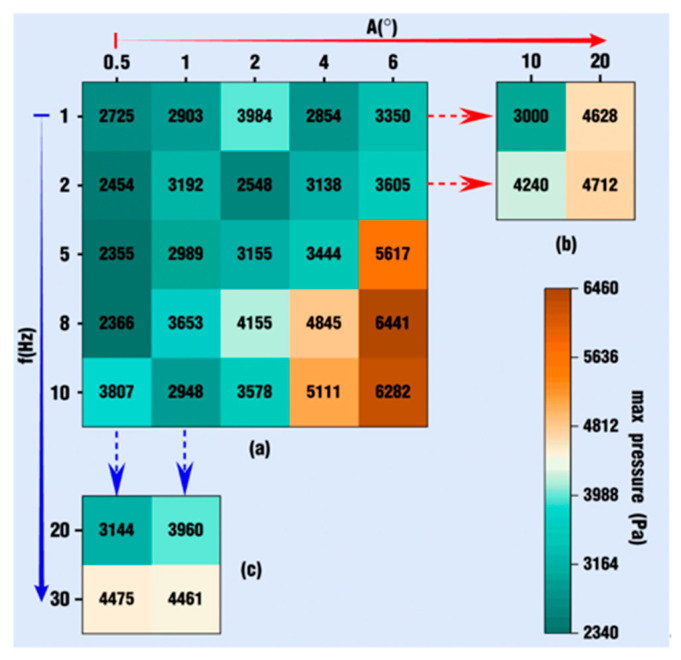
Max pressure of expanding cases: (**a**) normal conditions, (**b**) high amplitude and low frequency, and (**c**) low amplitude and high frequency.

At the same time, in order to analyze the influence of high amplitude with low frequency and high frequency with low amplitude, relatively large amplitudes of 10 and 20 degrees with smaller frequencies of 1 and 2 Hz are selected for the former, and relatively large frequencies of 20 and 30 Hz with smaller amplitudes of 0.5 and 1 degrees are selected for the latter. Consequently, there are 30 groups of amplitudes and frequencies to be analyzed in this numerical simulation. This fully demonstrates the advantages of numerical simulation relative to experimental research, which otherwise will require significant resource consumption and time costs. At the end of the simulation, the maximum velocity and pressure values of the flow field under the above vibration conditions are summarized and compared, respectively, as shown in Figure 24 and Figure 25. In particular, it should be noted that in order to directly show whether the physical parameters obtained in vibration cases are greater than those in case 0 or not, the maximum velocity and pressure values displayed in Figure 24 and Figure 25 have subtracted the corresponding values in case 0, which are 0.314 m/s for the max velocity and 4953 Pa for the max pressure, respectively. In other words, those numbers shown in the above two figures are relative values.

Figure 24 reveals the max velocity among various vibration cases at the end of the simulation. Figure 24a shows the expanded basic cases, Figure 24b shows the cases with high amplitudes and low frequencies, and Figure 24c shows the cases with low amplitudes and high frequencies. As a whole, all the values in Figure 24 are positive, indicating that the maximum velocity under vibration conditions can be obtained with greater values than those in case 0. Therefore, the superimposed vibration excitation strengthens the velocity field of the fluid flow. In Figure 24a, it can be observed that the maximum velocity values gradually increase along the diagonal direction of the entire table, and there is an almost nine-fold increase from 0.034 m/s for *f* = 1 Hz with *A* = 0.5 degrees to 0.338 m/s for *f* = 10 Hz with *A* = 6 degrees. However, if the observation is performed more carefully, it can be found that there is no definite change trend in the maximum velocity values in the table. When *f* = 5 Hz with *A* = 2 degrees, the maximum velocity is 0.072 m/s, which is less than the value of its previous case, *f* = 2 Hz, with *A* = 1 degree. In the horizontal direction, where the amplitudes are in an increasing trend or in the vertical direction and frequencies are in an increasing trend, there is also no ever-increasing or ever-decreasing trend for maximum velocity values.

Figure 24b shows the maximum velocity under the excitation condition of larger amplitudes and low frequencies. As for Figure 24b itself, the maximum velocity values increase with the addition of excitation amplitudes. However, if the values in Figure 24b are checked together with the data shown in Figure 24a, there is neither an increasing trend nor a decreasing trend. Figure 24c displays the maximum velocity under high-frequency and small-amplitude excitation. Obviously, the maximum velocity decreases when *A* = 0.5 degrees and increases significantly when *A* = 1 degree.

Figure 25 displays the max pressure among various expanding vibration cases at the end of the simulation. Similar to Figure 24, all of the values in Figure 25 are positive on the whole, indicating that the maximum pressure under vibration conditions can be obtained with greater values than those in case 0. Therefore, the superimposed vibration excitation could also strengthen the pressure field of the fluid flow. In Figure 25a, showing the expanded basic cases, it can be seen that the lower right corner is orange, and the top left corner is blue, indicating that the maximum pressure values gradually increase along the diagonal direction of the entire table. However, this trend is not consistent. When *f* = 1 Hz with *A* = 2 degrees, the maximum pressure is 3984 Pa, which is much larger than the values of its surrounding cases. When *f* = 10 Hz with *A* = 1 degree, the maximum pressure is 2948 Pa, which is significantly smaller than the values of its surrounding cases. Similarly, in the horizontal direction, with amplitudes increasing, or in the vertical direction, with frequencies increasing, the maximum pressure values do not always increase or decrease. Figure 25b shows the maximum pressures under the excitation conditions of larger amplitudes and low frequencies. As for Figure 25b itself, the maximum pressure values increase with the addition of excitation amplitudes. However, if the values in Figure 25b are compared with the data shown in Figure 24a, there is neither an increasing trend nor a decreasing trend. Figure 25c displays the maximum pressures under high-frequency and small-amplitude excitation. Obviously, at *A* = 0.5 degrees, the maximum pressures decrease when *f* = 20 Hz and increase when *f* = 30 Hz. However, at *A* = 1 degree, the maximum pressure values both increase when the frequency equals 20 Hz or 30 Hz.

From the above results and discussion on Figure 24 and Figure 25, it can be concluded that there is no obvious trend of a constant increase or a constant decrease with the addition of *VI* values. Some combination cases show a local upward trend, while others show a local downward trend. High frequencies with low amplitudes or great amplitudes with small frequencies cannot necessarily improve the maximum velocity in the flow field. There are some experiments [1,32,50] reported in the published literature that have proved this result. Wang et al. [50] studied the rheological behavior of polypropylene (PP)/CaCO_3_ compounds with the vibrating frequencies set to be 0, 4, 8, and 12 Hz and the vibrating amplitudes set to 0, 0.1, 0.15, 0.2, and 0.25 mm. The experimental results indicated that the apparent viscosity of compounds decreases as the amplitudes increase, while the viscosity first decreases and then rises as the frequency increases. By contrast, orthogonal experiments by Xue et al. [1] show that the mechanical properties of poly lactic acid/ethylene propylene diene monomer compounds reach the greatest value under a medium frequency (10 Hz) and amplitude (1.0 mm), and the tensile strength and elongation at break are increased by 65.34% and 82.92%, respectively. These influence trends of vibrating parameters on polymer properties are basically in agreement with the results obtained in this research.

With the analysis in the previous sections, it can be concluded that the superposition of the vibration field can gradually increase the velocity and pressure values as the *VI* value increases. The two conclusions may seem contradictory, but they are actually consistent. For example, the maximum velocity value in Figure 24a does increase with the addition of vibration amplitude or frequency in the previously selected cases. Therefore, the conclusions in this section greatly make up for the shortcomings of the previous analysis and make the research results more comprehensive.

To sum up, cases under different combinations of amplitudes and frequencies can obtain larger velocities and smaller pressures, smaller velocities and larger pressures, or both larger velocities and larger pressures. In actual polymer processing, different materials require different velocities and pressures due to their special properties. Take the PP/CaCO_3_ component, for example; the effect of vibration amplitude and frequency on the apparent viscosity of the lower filler content (3 wt%) is more obvious than that for the high filler content (20 wt%) [50]. Therefore, it is necessary to select an appropriate combination of the amplitude and frequency according to the actual properties of the processing materials and their processing requirements.

## 4. Conclusions

In this study, a numerical investigation using a meshless SPH simulation method is carried out to model the flow properties of fluids under a vibration system developed by our research team, and sinusoidal rotational speeds with various amplitudes and frequencies are loaded on the co-rotating twin screws of a conveying element. Specifically, the fully filled state is selected and analyzed in detail to reveal the effects of various superimposed excitation parameters on the flow properties of polymers and to determine whether a prior combination of amplitudes and frequencies exists for better flow properties. The following conclusions are drawn:

(1) From the perspective of the entire channel under specific pairs of amplitudes and frequencies, the superimposed vibration excitation leads to progressively higher velocity and pressure values as the *VI* value increases. Moreover, the velocity field demonstrates a more significant influence on increasing the number of crossover particles compared to the pressure field.
(2) Statistical analysis based on cross-sectional clips perpendicular to the *Z*-axis reveals that the amplitudes and frequencies of the superimposed excitation can positively influence particle distribution in certain regions of the flow channel. However, this effect is not sufficiently pronounced to be clearly discernible in other regions.
(3) Comparative results involving 400 tracked particles in each case, observed from the inter-meshing zone of the twin screws, exhibit distinct differences among cases with varying *VI* values. As the *VI* values listed in Table 1 increase, both velocity and pressure values become larger and more oscillatory, thereby enhancing the flow characteristics of particles within the channel.
(4) An expanded set of cases with various combinations of amplitude and frequency yields a more comprehensive conclusion: there is no consistent monotonic increase or decrease in velocity and pressure values with rising amplitudes and frequencies. While certain amplitude–frequency pairs exhibit a local upward trend, others show a local downward trend. Overall, cases with higher amplitudes and frequencies are capable of achieving greater velocity or pressure values, but such an outcome is not guaranteed when compared to cases with lower amplitudes and frequencies.

## Figures and Tables

**Table 1:** Simulation setting for steady and dynamic states.

Case No.	Excitation	*VI*	*f*	*A*	*ω*	Viscosity	*dp*	*dt*	$\rho_{0}$	*c*
		°/s	Hz	°	°/s	Pa.s	mm	s	kg/m^3^	m/s
0	false	0	0	0	360	1	0.25	3.75 × 10^−6^	1000	20
1	true	5	10	0.5	360 + 0.5sin (20πt)					
2	true	6	1	6	360 + 6sin (2πt)					
3	true	20	10	2	360 + 2sin (20πt)					
4	true	30	5	6	360 + 6sin (10πt)					
5	true	60	10	6	360 + 6sin (20πt)					

## Data Availability

The data that support the findings of this study are available from the corresponding author upon reasonable request.
